# A*phelenchus yinyuensis* n. sp. (Tylenchina: Aphelenchoididae) found in *Terminalia* sp. in China

**DOI:** 10.21307/jofnem-2020-085

**Published:** 2020-09-03

**Authors:** Gu Jianfeng, Munawar Maria, Yiwu Fang, Liu Lele, Xianfeng Chen, Bo Cai

**Affiliations:** 1 Technical Centre of Ningbo Customs (Ningbo Inspection and Quarantine Science Technology Academy), Ningbo, 315100, Zhejiang, P. R. China; 2 Laboratory of Plant Nematology, Institute of Biotechnology, College of Agriculture and Biotechnology, Zhejiang University, Hangzhou, 310058, Zhejiang, P. R. China; 3 Hainan Province Engineering Research Center for Quarantine, Prevention and Control of Exotic Pests, Haikou Customs District, Haikou, Hainan, P. R. China.

**Keywords:** DNA sequencing, Fungal feeder nematode, Morphology, New record, Phylogeny

## Abstract

During a nematode biodiversity survey in Hainan Province, China, *Aphelenchus yinyuensis* n. sp. was detected in the rhizosphere of *Terminalia* sp. It is characterized by medium-sized a body of adult nematodes, i.e. 793 (639-877) μm and 756 (647-863) μm for females and males, respectively, with low, rounded, not offset lip region. The lateral field has 10 incisures. The excretory pore is located posterior to the nerve ring. The vagina is not sclerotised and the vulva has simple lips without a flap. The PUS is well developed and forms *ca* 45 to 83% of the vulva to anus distance. Female tail is straight, cylindrical, ca 2.7 times longer than anal body diam, tail tip broad, and bluntly rounded. Males have four pairs of caudal papillae and spicule 28.7 (25.8-32.3) μm long in the chord and well developed bursa, extending to the tail tip. 18S and 28S rRNA phylogenetic analyses were performed for the new species, and the ITS analysis was not performed due to low posterior probability support. Phylogenetically, the new species grouped with *Aphelenchus avenae* and this is the first new *Aphelenchus* species ever described from China.

The Aphelenchoidea (Fuchs, 1937; Thorne, 1949) is a large group of stylet bearing nematodes that have adapted to a wide range of ecological relationships including phytoparasitism, predation, fungus feeding, association with insects in soil and obligate insect parasitism (Nickle, 1970). The majority of studied members are fungal feeders (Hunt, 1993). However, only *Bursaphelenchus* and *Aphelenchoides* species have gained much importance due to their parasitic potential and quarantine regulations. Species of genus *Aphelenchus* (Bastian, 1865) mostly occur in soil, leaf sheaths, plant crowns, and cortex of some roots where they presumably feed on fungal hyphae. The genus contains 11 nominal species (Hunt, 2008), only *A. avenae* attracted the economic value because it is a non-parasitic, fungivores nematode commonly found in croplands of all over the world and has been tested as a biological agent to control soil-borne plant pathogens (Azimi, 2018; Haraguchi and Yoshiga, 2020).

In an attempt to investigate the nematode biodiversity in Hainan Province, a population of *Aphelenchus* nematode was detected from *Terminalia* sp. in Sansha City in an unbalanced adult ratio, i.e. more males than females. The unbalanced adult ratio is not common in *Aphelenchus* species, in fact, the research has demonstrated that under unfavorable conditions the Pine Wood Nematode can regulate its population by changing sex ratios (Cui et al., 2018). Therefore, the isolated population was examined carefully and morpho-molecular characterization was performed. Our results confirmed that this population has unique characters and belonged to a new species; therefore, it is describe herein as *Aphelenchus yinyuensis* n. sp.

## Materials and methods

### Isolation and morphological observation of nematodes

Nematodes were extracted from soil and root samples using the modified Cobb sieving and flotation-centrifugation method (Jenkins, 1964). For morphometric studies, nematodes were killed and fixed in hot formalin (4% formaldehyde) and processed to ethanol-glycerin dehydration according to Seinhorst (1959) as modified by De Grisse (1969) and mounted on permanent slides. Measurements were made on mounted specimens and light micrographs and illustrations were produced using a Zeiss microscope equipped with a Zeiss AxioCam MRm CCD camera.

### Molecular analyses

DNA samples were prepared according to Li et al. (2008). Four sets of primers (synthesized by Invitrogen, Shanghai, China) were used in the PCR analyses to amplify sequences of the near full-length 18S region, D2-D3 expansion segments of 28S, and ITS of ribosomal RNA genes (rDNA). The 18S region was amplified with primers 988F/1912R and 1813F/2646R (Holterman et al., 2006). The 28S D2 to D3 region was amplified with primers D2A/D3B (De Ley et al., 1999), and the ITS was amplified using primers TW81/AB28 (Tanha Maafi et al., 2003). PCR conditions were as described by Ye et al. (2007) and Li et al. (2008). PCR products were separated on 1% agarose gels and visualized by staining with ethidium bromide and products of sufficiently high quality were purified for cloning and sequencing by Invitrogen, Shanghai, China.

### Phylogenetic analyses

The sequences of the near full-length 18S region and D2 to D3 expansion segments of 28S of *A. yinyuensis* n. sp. were compared with the species which shares the close phylogenetic relationship with *Aphelenchus s*pecies available in GenBank. The selected sequences were aligned by MUSCLE (Edgar, 2004). The GTR + I + G model was selected as the best-fit model of DNA evolution for both 18S, and 28S D2 to D3 regions using jModelTest2 (Darriba et al., 2012) according to the Akaike Information Criterion (AIC). The phylogenetic tree based on rDNA genes was obtained using MrBayes 3.2.6 (Ronquist and Huelsenbeck, 2003) with four chains (three heated and one cold). Model parameters were unlinked and the overall rate was allowed to vary across partitions. The number of generations for the total analysis was set to 1 × 107, with the chain sampled every 1,000 generations and a burn-in value of 25%. The 50% majority rule consensus phylogenetic consensus trees were visualized using FigTree v. 1.4.3 (Rambaut, 2016). The sequences divergence value was calculated by MEGA X under the p-distance and pairwise deletion settings (Kumar et al., 2018).

## Results

### Systematics

**Figure 1:**
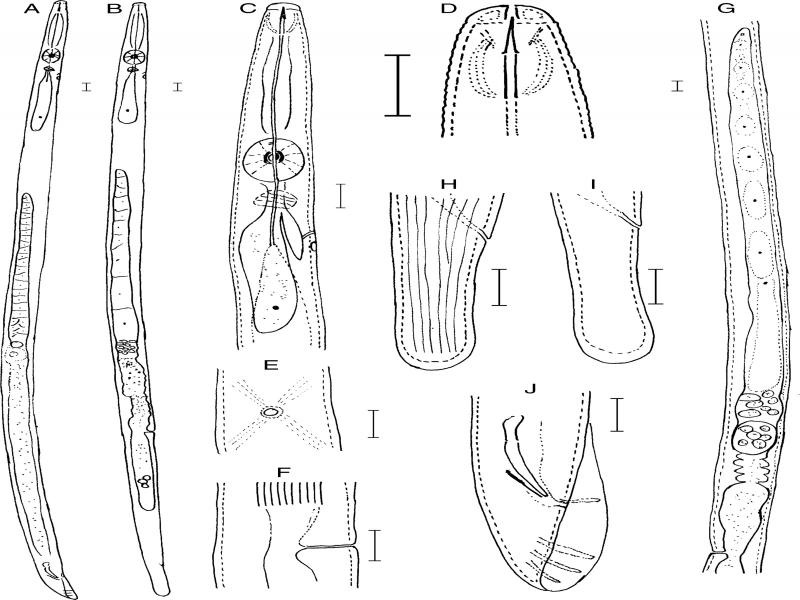
*Aphelenchus yinyuensis* n. sp. A: Entire male; B: Entire female; C: Anterior region; D: Lip region; E: Vulval region lateral view; F: Vulval region ventral view; G: Female reproductive system; H, I: Female tails; J: Male tail. Scale bars (A, B = 20* *μm; C-J = 10 μm).

**Figure 2:**
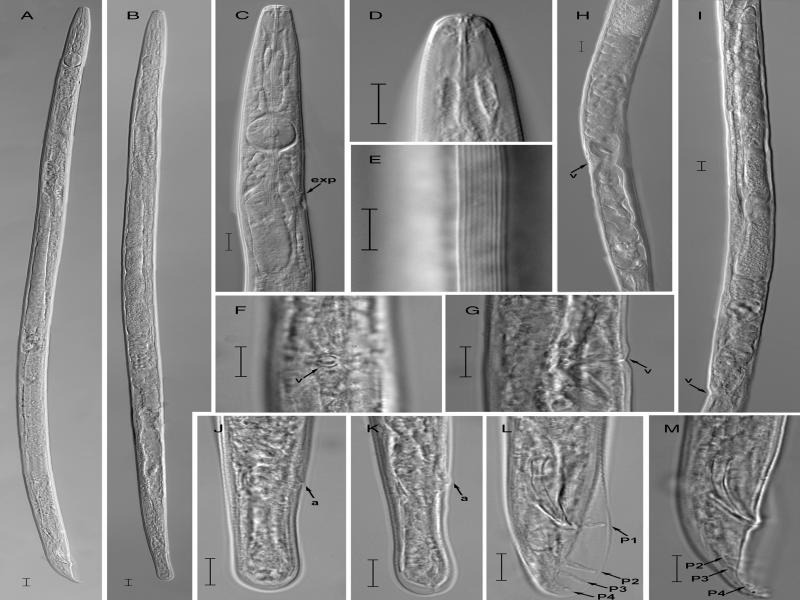
Light photomicrographs of *Aphelenchus yinyuensis* n. sp. A: Entire male; B: Entire female; C: Anterior region: D: Lip region; E: Lateral lines; F: Vulval region lateral view; G: Vulval region ventral view; H: Vulval region with post-uterine sac; I: Female reproductive system; J, K: Female tails; L, M: Male tail showing papillae arrangement (arrowheads). (ex = excretory pore; h = hemizonid; P + number = genital papillae; scale bars = A, B = 20* *μm; C-M = 10 μm).

**Figure 3:**
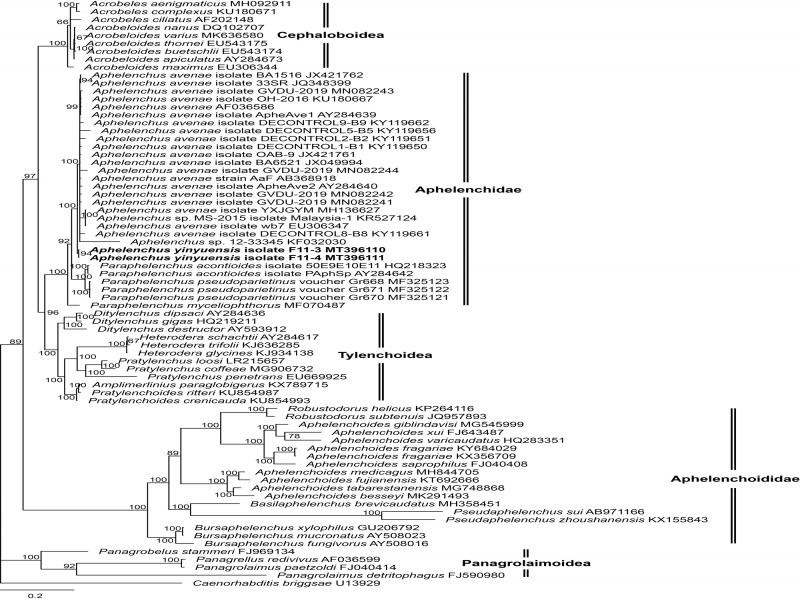
Phylogenetic relationship of *Aphelenchus yinyuensis* n. sp. and other nematodes based on near full length 18S of rDNA. Bayesian tree inferred from 18S under GTR + I + G model (lnL = −19,759.9398; freqA = 0.2462; freqC = 0.2010; freqG = 0.2710; freqT = 0.2818; R(a) = 1.1079; R(b) = 2.1894; R(c) = 1.2504; R(d) = 0.7956; R(e) = 3.9256; R(f) = 1.0000; Pinvar = 0.1260; Shape = 0.5890). Posterior probability values exceeding 50% are given on appropriate clades.

**Figure 4:**
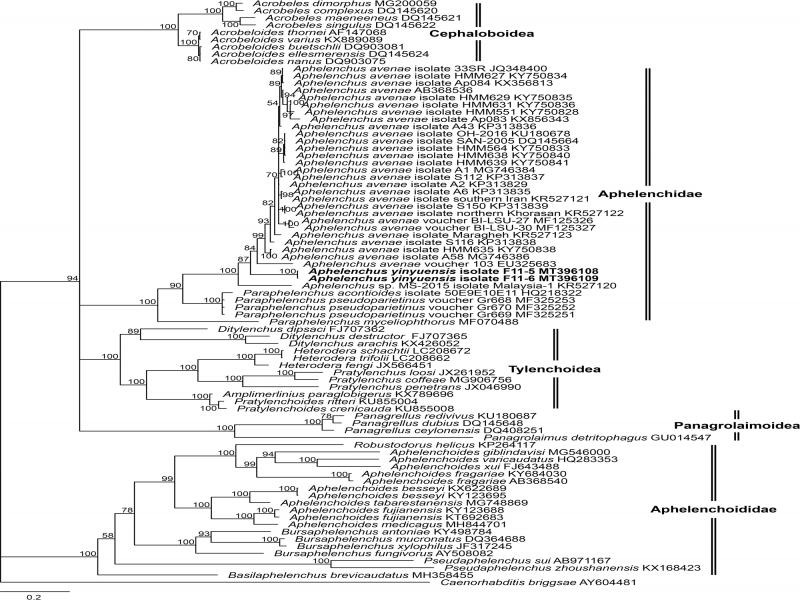
Phylogenetic relationship of *Aphelenchus yinyuensis* n. sp. and other nematodes based on 28S D2 to D3 of rDNA. Bayesian tree inferred from 28S D2 to D3 under GTR + I + G model (lnL = −17,543.4883; freqA = 0.1670; freqC = 0.1883; freqG = 0.3314; freqT = 0.3134; R(a) = 1.3541; R(b) = 3.5721; R(c) = 1.7006; R(d) = 0.5742; R(e) = 4.6218; R(f) = 1.0000; Pinvar = 0.1400; Shape = 0.6940). Posterior probability values exceeding 50% are given on appropriate clades.

*Aphelenchus yinyuensis* n. sp.

(Figs. 1-4, Table 1)

#### Description

##### Female:

The body of female is long, cylindrical, slightly ventrally arcuate or straight when heat relaxed. Cuticle is weakly annulated, lateral field with 10 incisures (i.e., 9 ridges) (reduced to 6 incisures at the tail end). Lip region is low, rounded, not offset. Stylet is 14.3 (13.2-15.2) µm long without basal swelling, conus forming ca two-fifths of total length. Procorpus is cylindrical ca 3-4 times stylet length. Median bulb (metacorpus) is well developed, ovoid, and conspicuous valve plates situated centrally. Dorsal esophageal gland orifice opening into lumen of median bulb mid-way between anterior end of metacorpal valve and anterior end of median bulb. Esophago-intestinal junction located 20 to 25 µm posterior to base of metacorpus, 91 to 114 µm from the anterior end. Esophageal gland lobe is slender, ca 3 times body diam., overlapping intestine dorsally. Nerve ring is located ca one metacorpal valve length posterior to metacorpus. Excretory pore is located posterior to the nerve ring. Hemizonid is slightly posterior to excretory pore, but sometimes at the same level of excretory pore. Reproductive system is monodelphic, prodelphic, occupying ca 45 to 55% of body length, consisting of ovary, oviduct, spermatheca, crustaformeria, uterus, vagina and post-uterine sac (PUS), with developing and developed oocytes arranged in a single row in anterior part and posterior part of ovary. Oviduct is comparatively longer, connecting ovary and spermatheca. Spermatheca is round or oblong, containing round sperm, present in majority of specimens. Uterus is thick-walled. Vagina wall is parallel not sclerotised, vulva pore shape, and lips simple without flap. In some specimens, vulval region appears sunken. PUS is well developed, extending for ca 44.5 to 82.7% of vulva to anus distance. Rectum and anus are visible. Tail is straight, cylindrical, ca 2.7 times longer than anal body diam. Tail tip is broad and bluntly rounded.

##### Male:

The body of male is cylindrical, posterior region slightly curved, Cuticle, and anterior region similar to female. Testis is outstretched, *ca* 60 to 89% of body length, developing spermatocytes arranged in one row, well developed spermatids arranged as two rows. Spicules are paired, slender, and ventrally arcuate and slightly cephalated proximally, 28.7 (25.8-32.3) μm long in chord. Gubernaculum is linear. Bursa is well developed, extending from the proximal region of the spicules to the tail tip and supported by four pairs of bursal papillae (ribs), one pair preanal, and the other three near the tail end. Tail is short, conoid, tapering to a pointed terminus.

##### Type habitat and locality:

This population was found in the rhizosphere of *Terminalia* sp. from Yinyu Islet, Yongle Islands, Sansha City, Hainan Province, China (GPS: The geographical position of the sampling site is: 16°35′03″N, 111°42′39″E. Date: September 23, 2019. Collectors: Bo Cai, Rui Meng).

##### Type material:

Holotype female, 12 female and 14 male paratypes (slide numbers F11-1 to F11-9) were deposited in the nematode collection of Ningbo Customs Technical Center, China. Three paratype females and one paratype male (T572) deposited in the Canadian National Collection of Nematodes, Ottawa, Canada.

##### Etymology:

The species is named after the type locality.

##### Diagnosis and relationships:

*Aphelenchus yinyuensis* n. sp. is characterized by the female body length of 793 (639-877) μm and male length of 756 (647-863) μm. Lip region is low, rounded, not offset. The lateral field has 10 incisures. The slender stylet is 14.3 (13.2-15.2) μm long and has no basal swellings. The excretory pore is located posterior to the nerve ring. The vagina is not sclerotised and the vulva has slightly protruding lips and lacks a flap. The PUS is well developed and forms ca 45 to 83% of the vulva to anus distance. Female tail is straight, cylindrical, ca 2.7 times longer than anal body diam., tail tip broad and bluntly rounded. Male testis is outstretched, ca 60 to 89% of body length. Spicules are paired, slender and ventrally arcuate, 28.7 (25.8-32.3) μm long in the chord. Gubernaculum is linear. Bursa is well developed, extending from the proximal region of the spicules to the tail tip, and supported by four pairs of bursal papillae (ribs).

Up to now, genus *Aphelenchus* contains 11 nominal species (Hunt, 2008), of which very few are adequately characterized to allow reliable identification, except the type species, *A. avenae*. The other 10 species are *A. amorphophallusi* (Singh and Agrawal, 1976); *A. bastiani* (Shavrov, 1967b); *A. eremitus* (Thorne, 1961); *A. isomerus* (Anderson and Hooper, 1980); *A. kralli* (Samibaeva, 1966); *A. mirzai* (Das, 1960); *A. paramonovi* (Nesterov and Lisetskaya, 1965); *A. siddiqii* (Kumar, 1982); *A. sparsus* (Thorne and Malek, 1968); and *A. tumidicaudatus* (Shavrov, 1967a).

Another species i.e. *A. assamensis* (Chanu et al., 2017) was described from India may not belong to the *Aphelenchus* and have mistakenly described under *Aphelenchus*. The male of *A. assamensis* does not have gubernaculum and bursa, and additionally, the spicule shape is not typical of the genus.

The new species differs from *A. avenae* by a smaller V value (72.8 (65.2-77.6) vs 76.5 (74-78)), smaller female c value (21.0 (16.7-27.1) vs 31(27-35)), smaller c’ value (2.7 (2.3-3.8) vs 1.75 (1.5-2.5)), vulva shape (pore shape *vs* a short transverse oval) and number of lateral line (10 vs 10-14).

It differs from *A. amorphophallusi* by shorter female body length (793 (639-877) vs 960 (800-1080) µm), shorter stylet length (14.3 (13.2-15.2) vs 16 (15-17) µm), smaller V value (72.8 (65.2-77.6) vs 77 (75-77.8)), smaller c value (21.0 (16.7-27.1) vs 29.4 (25.3-36)), by lateral lines (10 vs 8), and excretory pore position (posterior to the nerve ring vs anterior to the nerve ring).

It is different from *A. eremitus* by smaller V value (72.8 (65.2-77.6) vs 81), by number of lateral lines (10 vs 8), longer tail length (2.3-3.8 times anal body diameter vs slightly longer than anal body diameter), position and number of bursal ribs (4 pairs vs 3, first pair preanal vs opposite anus), and bigger male T value (70.5 (59.4-89.0) vs 50).

The new species differs from *A. tumidicaudatus* by number of lateral lines (10 vs 12), smaller a value (30.5 (26.9-33.6) vs 37.9-44.2), smaller c value (21.0 (16.7-27.1) vs 27.8-32.3), PUS length (60.9 (44.5-82.7) % of the vulva to anus distance vs about 1/4 of the vulva to anus distance (measured from the original drawing)), and tail shape (cylindrical, with broad and bluntly rounded tip vs inflated tail tip).

The new species can be distinguished from *A. kralli* by longer female body length (793 (639-877) vs 560 µm), smaller V value (72.8 (65.2-77.6) vs 76.1), smaller a value (30.5 (26.9-33.6) vs 45), tail length and shape (ca 2.7 times longer than anal body diam., tail tip broad and bluntly rounded vs slightly longer than anal body diam., lobed, dorsal and ventral margins spike like).

It differs from *A. sparsus* by bigger a value (30.5 (26.9-33.6) vs 25), smaller V value (72.8 (65.2-77.6) vs 76), number of lateral lines (10 vs 4-6), smaller c value (21.0 (16.7-27.1) vs 34), PUS length (60.9 (44.5-82.7) % of the vulva to anus distance vs about as long as body width), and tail length (ca 2.7 times longer than anal body diam., vs about 1 time anal body width (measured from the original drawing).

The new species differs from *A. mirzai* by longer female body length (793 (639-877) vs 570-690 µm), excretory pore position (107 (96.6-123.3) µm from the anterior end, slightly posterior to the nerve ring vs 130 µm from the anterior end, about 20 to 30 posterior to nerve ring), longer male body length (756 (647-863) vs 570 µm), smaller a value of males (31.9 (26.7-37.0) vs 48.3), longer spicule length (28.7 (25.8-32.3) vs 22 µm) and number of caudal papillae (4 pairs vs 3 pairs).

It differs from *A. siddiqii* by lip region not set off vs set off, shorter female body length (793 (639-877) vs 930 (730-1030 µm), shorter stylet length (14.3 (13.2-15.2) vs 17.2 (16.7-18.0) µm), smaller c value (21.0 (16.7-27.1) vs 33.5 (27.1-43.0), number of lateral lines (10 vs 12), longer PUS length (100.7 (66.3-120.2) vs 60 µm), longer tail length (ca 2.7 times longer than anal body diam. vs 1-1.5 times); bigger a value of males (31.9 (26.7-37.0) vs 24.8), shorter spicule length (28.7 (25.8-32.3) vs 35 µm), and shorter stylet length (13.8 (12.2-15.7) vs 17 µm).

*A. yinyuensis* differs from *A. paramonovi* by smaller a value (30.5 (26.9-33.6) vs 45), longer PUS length (60.9 (44.5-82.7) % of the vulva to anus distance vs about 20% of the vulva to anus distance (measured from the original drawings), and longer tail length (ca 2.7 times longer than anal body diam. vs 1-1.5 times (measured from the original drawings); anal region smooth from the body contour vs swollen.

Finally, it differs from *A. isomerus* by smaller c value (21.0 (16.7-27.1) vs 28 (22-32), bigger c’ value (2.7 (2.3-3.8) vs 1.9 (1.6-2.2), smaller V value (72.8 (65.2-77.6) vs 77(75-78), longer tail length (38.4 (24.2-44.9) vs 25(20-29) µm), and number of lateral lines number (10 vs at least 14).

Furthermore, the new species differs from all other known species by having an unbalanced adult ratio, i.e., more males than females, while in all other described species males were not found or rare. In addition, the PUS of *A. yinyuensis* n. sp. is quite long, well developed and filled with sperm, while the PUS in other *Aphelenchus* species is usually short, collapsed, or shrink, without sperm.

### Molecular characterization and phylogeny

The sequences of nearly full-length 18S (1707 bp, GenBank accession numbers MT396110- MT396111), 28S D2-D3 region (772 bp, MT396108-MT396109) and ITS region of rDNA (661 bp, MT396103-MT396104) of *A. yinyuensis* n. sp. were obtained. Phylogenetic relationships among the isolates were determined separately for each dataset using Bayesian inference (BI), with *Caenorhabditis briggsae* (Dougherty and Nigon, 1949), as an outgroup for the rDNA (18S and 28S D2-D3 region) datasets. The sequences were aligned by MUSCLE and modified in datasets of 1850 characters for 18S and 853 characters for 28S D2-D3.

The Bayesian phylogenetic tree of the 18S gene represented a high support (Posterior Possibility PP = 100) clade of Aphelenchidae (Fuchs, 1937) which is consisted of two genera, *Aphelenchus* and *Paraphelenchus* (Micoletzky, 1922). The clade of Aphelenchidae showed a sister phylogenetic relationship with the clade of Tylenchoidea Örley, 1880 and clustered with Cephaloboidea Filipjev 1934 into a big clade. And this clade is sister to the clade of family Aphelenchoididae (Skarbilovich, 1947) which is another member of superfamily Aphelenchoidea (Fuchs, 1937). The new species occupies as an early branch of *Aphelenchus* clade. The sequence divergence of *A. yinyuensis* n. sp. and different populations of *A. avenae* which is the only molecularly characterized valid species in this genus range from 0.52% (5/967 bp JX049994) to 2.23% (20/896 bp KY119651).

In the 28S D2-D3 gene tree, *Aphelenchus* and *Paraphelenchus* still formed a high support (Posterior Possibility PP = 100) clade which is congruent with the 18S gene tree. However, the Aphelenchidae clade embedded in a big clade with Tylenchoidea clade, Cephaloboidea clade, and Panagrolaimoidea (Thorne, 1937) clade and demonstrated paraphyletic relationships. The new species clustered with other *Aphelenchus* species as an independent branch. The sequence divergence of *A. yinyuensis* n. sp. and different populations of *A. avenae* ranged from 4.20% (14/333 bp MF125326) to 16.28% (78/479 bp KX856343).

No phylogenetic tree based on the ITS sequence was carried out in the present study since the high sequence divergences and lack of support in the posterior probabilities among related species. However, the sequence divergence of *A. yinyuensis* n. sp. and different populations of *A. avenae* were still calculated and ranged from 16.23% (106/653 bp AF119048) to 18.10% (118/652 bp KF032030). The phylogenetic trees and molecular characterized analysis strongly supported the status of *A. yinyuensis* n. sp. as a new species.

## Discussion

Morphologically, the *Aphelenchus* species presents unique characters, e.g. number of lateral lines are not fixed, from 4 (*A. sparsus*) to as many as 14 (*A. isomerasus*). The tail is typical for all the species except *A. eremitus* (obtuse), *A. krailli* (lobed with spiked dorsal and ventral margins), *A. paramonovi* (bulged out at anus and narrows down to a rounded terminus), *A. sparsus* (squarish bluntly rounded) and *A. tumicaudatus* (rounded with a notched ventral side).

The genus *Aphelenchus* is poorly characterized in terms of molecular characterization. Majority of the previously described species, following their formal description, are seldom mentioned again in the scientific literature. DNA sequence-based studies were only carried out for *Aphelenchus avenae*; therefore, the new species grouped with it in our phylogenetic analysis. To get better insights of *Aphelenchus* species relationship, it is recommended to perform a more extensive phylogenetic survey with a better taxon sampling and geographic representation.

The *Aphelenchus* species are reported from temperate, subtropical and tropical regions of the world, many populations are lacking male and most species considered as parthenogenetic (Manzanilla-Lopez and Marban-Mendoza, 2012). However, *A. yinyuensis* n. sp. found during this study has a considerable number of males, an unbalanced sex ratio could be due to the stress. Studies have indicated that *Terminalia* species produce different secondary metabolites (Lee et al., 2007; Eloff et al., 2008; Pfundstein et al., 2010; Fahmy et al., 2015). To this point, we speculate that an unbalanced sex ratio might be due to the host and further research is required to explore this aspect of nematode biology.

**Table 1.** Morphometrics of *Aphelenchus yinyuensis* n. sp.

	Female		Male
Character	Holotype	Paratypes	Paratypes
n	–	15	15
L	792	793 ± 71.9 (639-877)	756 ± 59.6 (647-863)
a	31.0	30.5 ± 2.7 (26.9-33.6)	31.9 ± 3.4 (26.7-37.0)
b	9.6	7.7 ± 1.0 (6.2-8.8)	7.5 ± 0.7 (6.1-8.7)
b′	5.0	4.8 ± 0.6 (3.9-5.7)	4.5 ± 0.4 (3.8-5.3)
c	19.5	21.0 ± 3.1 (16.7-27.1)	26.7 ± 2.6 (22.1-30.4)
c′	3.1	2.7 ± 0.4 (2.3-3.8)	1.8 ± 0.2 (1.5-2.2)
V or T	72.7	72.8 ± 3.8 (65.2-77.6)	70.5 ± 7.1 (59.4-89.0)
Lip region height	3.1	3.6 ± 0.3 (3.1-4.2)	3.4 ± 0.5 (2.5-4.2)
Lip region width	8.3	8.8 ± 0.5 (8.1-9.5)	8.5 ± 0.5 (7.8-9.5)
Stylet length	13.7	14.3 ± 0.7 (13.2-15.2)	13.8 ± 1.0 (12.2-15.7)
Body diam.	25.5	26.2 ± 3.1 (19.2-31.7)	23.9 ± 2.1 (19.2-28.2)
Median bulb width	15.3	16.1 ± 1.5 (13.1-19.6)	13.8 ± 1.0 (12.2-15.7)
Median bulb length	22.5	22.3 ± 1.8 (18.6-24.5)	20.1 ± 1.2 (17.8-21.9)
Median bulb length/diam. Ratio	1.5	1.4 ± 0.1 (1.2-1.5)	1.5 ± 0.1 (1.3-1.7)
E. pore from anterior end	113.6	107.9 ± 7.2 (96.6-123.3)	106.5 ± 7.2 (91.5-116.7)
Ovary length or Testis	436.3	407.4 ± 50.2 (280.2-458.3)	531.4 ± 54.1 (452.6-610.9)
Post-uterine sac	112.1	100.7 ± 15.5 (66.3-120.2)	–
Vulva to anus distance	176.3	166.9 ± 22.3 (131.2-195.0)	–
Post-uterine sac length/vulva to anus (%)	63.6	60.9 ± 10.4 (44.5-82.7)	–
Anal (cloacal) body diameter	13.1	14.2 ± 2.0 (10.6-17.3)	15.4 ± 1.0 (13.9-17.3)
Tail length	40.6	38.4 ± 5.9 (24.2-44.9)	28.4 ± 2.4 (23.6-32.2)
Spicule (curved median line)	–	–	28.0 ± 2.0 (24.3-31.3)
Spicule (Chord)	–	–	28.7 ± 1.7 (25.8-32.3)
Gubernaculum	–	–	13.2 ± 0.4 (12.2-13.7)
Hemizonid	115.3	110.1 ± 5.5 (100.1-117.3)	108.5 ± 7.3 (96.1-121.3)
Head to pharyngo-intestinal junction	92.9	103.2 ± 6.9 (91.0-113.5)	100.6 ± 5.5 (86.0-109.3)
Head to pharyngeal gland end	159.6	163.9 ± 12.1 (139.6-181.0)	165.9 ± 12.0 (149.3-188.2)
Vulva to tail terminus	203.1	213.1 ± 28.6 (156.3-251.8)	–

**Note:** All measurements are in µm and in the form: mean ± s.d. (range).
